# Laminar Subnetworks of Response Suppression in Macaque Primary Visual Cortex

**DOI:** 10.1523/JNEUROSCI.1129-20.2020

**Published:** 2020-09-23

**Authors:** Tian Wang, Yang Li, Guanzhong Yang, Weifeng Dai, Yi Yang, Chuanliang Han, Xingyun Wang, Yange Zhang, Dajun Xing

**Affiliations:** State Key Laboratory of Cognitive Neuroscience and Learning & IDG/McGovern Institute for Brain Research, Beijing Normal University, Beijing, 100875, China

**Keywords:** cortical layers, Macaque monkey, neural dynamics, primary visual cortex, suppression

## Abstract

Cortical inhibition plays an important role in information processing in the brain. However, the mechanisms by which inhibition and excitation are coordinated to generate functions in the six layers of the cortex remain unclear. Here, we measured laminar-specific responses to stimulus orientations in primary visual cortex (V1) of awake monkeys (male, *Macaca mulatta*). We distinguished inhibitory effects (suppression) from excitation, by taking advantage of the separability of excitation and inhibition in the orientation and time domains. We found two distinct types of suppression governing different layers. Fast suppression (FS) was strongest in input layers (4C and 6), and slow suppression (SS) was 3 times stronger in output layers (2/3 and 5). Interestingly, the two types of suppression were correlated with different functional properties measured with drifting gratings. FS was primarily correlated with orientation selectivity in input layers (*r* = −0.65, *p* < 10^−9^), whereas SS was primarily correlated with surround suppression in output layers (*r* = 0.61, *p* < 10^−4^). The earliest SS in layer 1 indicates the origin of cortical feedback for SS, in contrast to the feedforward/recurrent origin of FS. Our results reveal two V1 laminar subnetworks with different response suppression that may provide a general framework for laminar processing in other sensory cortices.

**SIGNIFICANCE STATEMENT** This study sought to understand inhibitory effects (suppression) and their relationships with functional properties in the six different layers of the cortex. We found that the diversity of neural responses across layers in primary visual cortex (V1) could be fully explained by one excitatory and two suppressive components (fast and slow suppression). The distinct laminar distributions, origins, and functional roles of the two types of suppression provided a simplified representation of the differences between two V1 subnetworks (input network and output network). These results not only help to elucidate computational principles in macaque V1, but also provide a framework for general computation of cortical laminae in other sensory cortices.

## Introduction

The laminar structure of the cerebral cortex is a common anatomic feature in the brain (Schroeder et al., 1998). The cortex has six cell layers with distinct intralaminar and interlaminar connectivity patterns (Lund, 1988; Callaway, 1998; Sincich and Horton, 2005). Consequently, cortical layers have different functional properties (Leventhal et al., 1995; Martinez et al., 2002; Buffalo et al., 2011; Goense et al., 2012; Self et al., 2013; Smith et al., 2013; van Kerkoerle et al., 2017; Bijanzadeh et al., 2018). However, the mechanisms by which different layers gain distinct functions by dynamically combining excitation and inhibition remain unclear (Hirsch and Martinez, 2006; Adesnik and Naka, 2018).

The goal of the current study was to reveal inhibitory effects and their relationships with functional properties throughout the depth of the macaque primary visual cortex (V1) and across V1 laminae. V1 has well-studied local laminar connections (Lund, 1988; Callaway, 1998; Sincich and Horton, 2005), which are assumed to be similar to laminar cortical circuitry in other cortical regions (Schroeder et al., 1998; Schroeder and Foxe, 2002; Linden and Schreiner, 2003). V1 layers 4C and 6, as input layers, receive excitatory drives from LGN and send excitatory signals to V1 output layers, layers 2/3 and 4B, after local intracortical processing. V1 output layers have strong horizontal connections and feedback connections (Rockland and Pandya, 1979; Stettler et al., 2002; Lund et al., 2003). The functional properties of cells in different V1 layers are markedly different (Hawken et al., 1988; Sato et al., 1996; Ringach et al., 2002; Gur et al., 2005; Yeh et al., 2009), reflecting different combinations of layer-specific inhibition and excitation (Xing et al., 2012; Bijanzadeh et al., 2018). Cortical inhibition plays important roles in the functional properties of V1, such as selectivity for stimulus orientation, size, luminance, and spatial frequency (Bredfeldt and Ringach, 2002; Tucker and Fitzpatrick, 2006; Liu et al., 2011; Xing et al., 2011; Adesnik et al., 2012). It has been reported that multiple forms of inhibitory effects exist in V1 (Ringach et al., 2003; Silberberg and Markram, 2007; Lee et al., 2012; Wilson et al., 2012), potentially because of unique neural circuitries (Adesnik et al., 2012; Bijanzadeh et al., 2018). However, the functional roles of inhibitory effects in macaque V1 are still largely unknown, or under debate (Mazer et al., 2002; Shapley et al., 2003; Goris et al., 2015). Unlike previous studies in rodents (Isaacson and Scanziani, 2011; Liu et al., 2011) and cats (Anderson et al., 2000; Martinez et al., 2002; Priebe and Ferster, 2006), directly measuring inhibition in V1 layers in monkey cortex is technically difficult. This difficulty leads to a lack of information about the laminar distribution of inhibition and their relationships with the functional properties of Monkey V1 layers.

To understand the different types of suppression across the layers of macaque V1, we activated V1 by rapidly flashing grating patches at different orientations and simultaneously recorded local field potentials (LFPs) and spiking activity in all layers of awake macaque V1. We then reconstructed the temporal development of orientation selectivity across layers. Benefitting from differences in time course and orientation selectivity between excitation and two types of suppression (Ringach et al., 2003; Xing et al., 2005), we distinguished spatiotemporal responses across V1 layers and found distinct laminar patterns for two types of suppression. To further investigate the functional roles of the two types of suppression, two important functional properties, orientation selectivity (Ringach et al., 2002; Gur et al., 2005) and surround suppression (Shushruth et al., 2009; Henry et al., 2013), were measured using a drifting grating stimulus. Interestingly, the two types of suppression were significantly correlated with orientation selectivity and surround suppression in a laminar-specific manner.

## Materials and Methods

### 

#### 

##### Preparation of awake monkeys

All procedures were conducted in compliance with the National Institutes of Health *Guide for the care and use of laboratory animals*, and were approved by the Institutional Animal Care and Use Committee of Beijing Normal University. Four male adult rhesus monkeys (DD, DY, DQ, and DK, *Macaca mulatta*, 5-7 years old, 6–8 kg) were used. Under general anesthesia induced with ketamine (10 mg·kg^−1^) and maintained with isoflurane (1.5%–2.0%), a titanium post was attached to the skull with bone screws for immobilizing the animal's head during behavioral training. After the animal had been trained in a simple fixation task, a circular titanium chamber (20 mm in diameter) with a removable lid was fixed over the craniotomy (15 mm anterior to the occipital ridge and 14 mm lateral from the midline), with dental cement for chronic recordings from V1. Antibiotics and analgesics were used after the surgery.

##### Behavioral task

A trial began when a monkey began fixating on a 0.1° fixation point (FP) presented on a CRT screen. In each trial, the FP was displayed in the center of the screen. The animal's eye positions were sampled at 120 Hz using an infrared tracking system (ISCAN). Within 300 ms after FP presentation, the animal was required to fixate within an invisible circular window (between 0.6° and 1° in radius) around the FP. After the animal maintained fixation for 100–400 ms (∼200 ms in most cases), the stimulus was displayed for 2–4 s (dependent on stimulus type), followed by a blank interval of 300 ms. The FP then disappeared, and the animal received a drop of water as reward. A trial was aborted if the animal's fixation moved outside the fixation window.

##### Electrophysiological recording

We simultaneously recorded neuronal activity from different layers in V1 using a linear array (U-probe, Plexon; 24 recording channels spaced 100 μm apart, each 15 μm in diameter). The linear array was controlled by a microelectrode drive (NAN Instruments), and the depth of each probe placement was adjusted to extend through all V1 layers. Raw data were acquired with a 128-channel system (Blackrock Microsystems). The raw data were high-pass filtered (seventh-order Butterworth with 1000 Hz corner frequency), and multiunit spiking activities (MUAs) were detected by applying a voltage threshold with a signal-to-noise ratio of 5.5. Single-unit activities (SUAs) were detected by offline spike sorting. Spike waveforms were carefully verified using custom spike sorting software (Yeh et al., 2009; Xing et al., 2010). Criteria for single units included a fixed shape of the action potential and the absence of spikes during the absolute refractory period. The raw data were also low-pass filtered (seventh-order Butterworth with 300 Hz corner frequency) to obtain LFPs. SUAs, MUAs, and LFPs were all downsampled to 500 Hz.

##### Visual stimulation

Visual stimuli were generated with a stimulus generator (ViSaGe; Cambridge Research Systems) under the control of a PC running a custom C++ program developed in our laboratory. The stimuli were displayed on a 22-inch CRT monitor (Dell, P1230, 1200 × 900 pixels, mean luminance 45.8 cd/m^2^, 100 Hz refresh rate). The typical viewing distance was 114 cm (with seven exceptions: the viewing distance was 57 cm for six recording session and 80 cm for one session). Three types of stimuli were used. Sparse noise was used to simultaneously map receptive fields (RFs). Random orientation presentation was used to measure orientation dynamics, align laminar positions, and check the verticality of the probe. Drifting grating stimuli were used to measure surround suppression and orientation selectivity.

##### RF mapping

After manually mapping the RFs of recording channels, we used sparse noise (Jones and Palmer, 1987) to identify the precise RF center. The sparse noise consisted of a sequence of randomly positioned (usually on a 13 × 13 or 11 × 11 sample grid) dark and bright squares (0.1°-0.3°, contrast 0.9°) against a gray background (luminance 45.8 cd/m^2^). Each sparse noise image appeared for 20 ms and with at least 50 repetitions. The sequence was cut into small segments based on trial length. We obtained a two-dimensional map of each channel. Responses averaged from *x* and *y* axes of each map were fitted with a one-dimensional Gaussian function to estimate the center position and radius of each RF (σ of Gaussian function). RFs were located within 5° of the fovea.

##### Orientation dynamics

**Figure 1.**
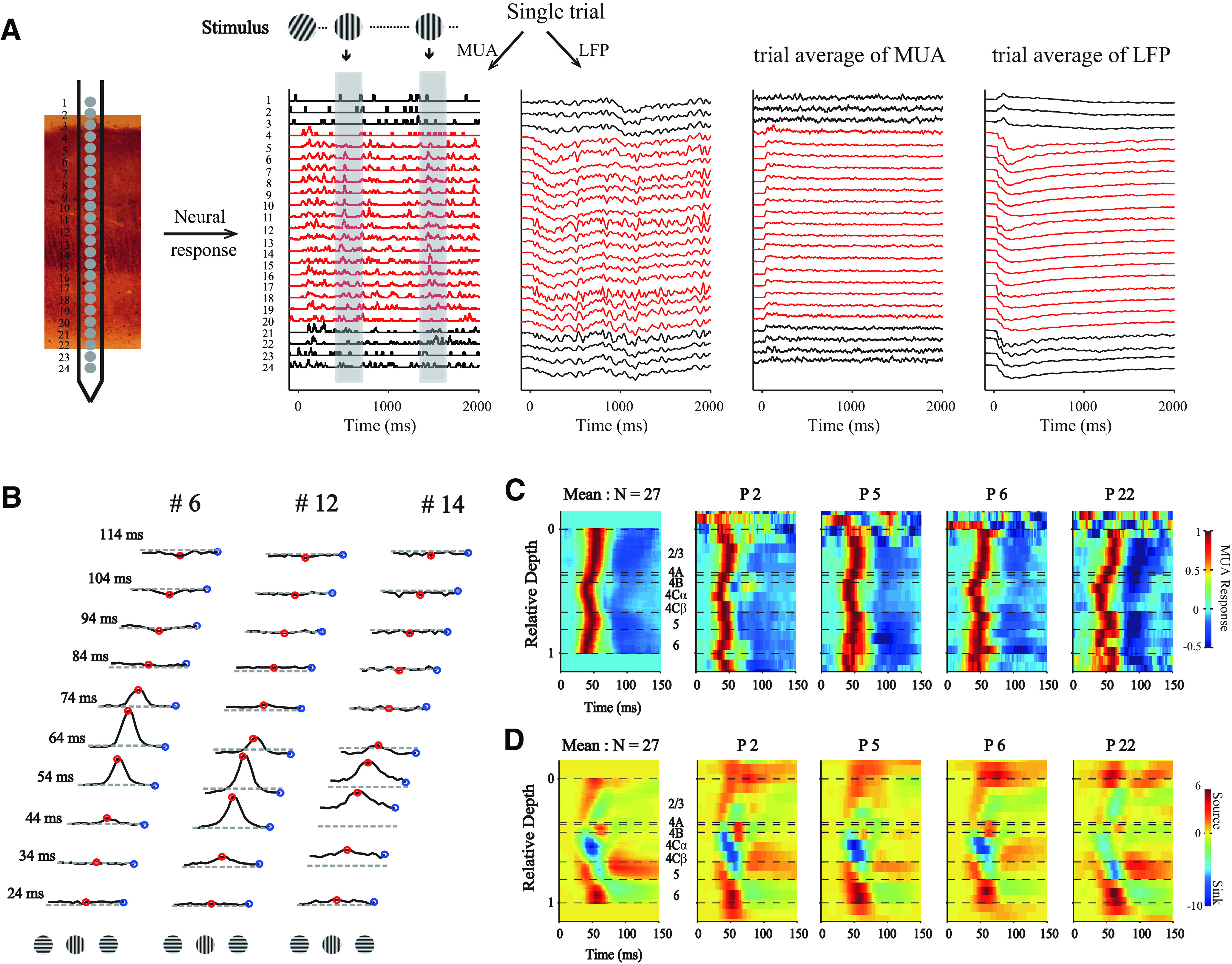
Simultaneous recordings of multiple sites throughout V1 layers. ***A***, Methods for laminar recording and reverse correlation. Left, Neural activity was recorded with U-Probe (Plexon, 24 channels, interchannel spacing 100 μm). The linear array was positioned vertically through the full depth of V1. Right, Demonstration of single trial and trial averages for MUA and LFP. Stimuli with different orientations were flashed for 20 ms in a random sequence. Shaded area represents the time window (−50 to 250 ms) for the triggered average. The neural activity of each channel was recorded with 2 ms resolution. Red represents sites within V1. ***B***, Dynamics of orientation tuning of the MUA at three example sites at different cortical depths from the probe placement in ***A***. Tuning curves were plotted every 10 ms, starting at 24 ms after stimulus onset and ending at 114 ms after stimulus onset. Red points represent the responses of the site to orientation at 0° (its preferred orientation). Blue points represent the responses of cells to orientation at 90° (orthogonal to preferred orientation). The tuning curves of each site were shifted, so that the preferred orientation was set to 0°. Dashed lines indicate the responses to a blank stimulus. ***C***, Laminar pattern of MUA from 1 animal (DD). For each probe placement (P), the averaged responses of MUA to all orientations were calculated. Patterns in first column were averaged from all probe placements in this animal (*N* = 27). The relative cortical depth was determined by signatures of MUA and CSD (see Materials and Methods). Horizontal black dashed lines indicate the laminar boundaries. ***D***, Similar to ***C***, but for CSD of the same probe placements. Each CSD pattern was normalized by its SD.

After the RF mapping experiment, a sequence of random flashed gratings with different orientations (random orientation experiment) was used to measure dynamic responses to orientations. Sinusoidal gratings of 18 different orientations equally spaced from 0° to 180°, plus “blanks” (defined as uniform frames with the same luminance as the mean luminance of the grating images; 10% or 20% of all stimuli) were used. For each orientation, the spatial phase was also varied: each orientation in the set was presented at eight different spatial phases, equally spaced from 0° to 360°. The size of the grating was 0.5°–2.5° in radius (at least 4 times larger than the RF of layer 4Cα for most probe placements, in 9 probe placements, the stimulus size was set at 2.5–4 times larger than the RF size of 4Cα), fixed within each session. We set the stimulus sizes to be at least 2.5 times larger than RFs of recorded sites to activate both local and global neural mechanisms in V1. Other parameters (2 cycles/deg for spatial frequency and 90% for contrast) of the gratings were fixed in all sessions. All of the gratings and blanks were randomly chosen and consisted of a sequence. Each stimulus in a sequence was randomly chosen and flashed for 20 ms with at least 50 repetitions (repetition varies from 50 to 300 between recording sessions). The sequence was cut into small segments based on trial length (2.2–4 s, with 110–200 stimuli). Each trial displayed one segment until all segments were used. Figure 1*A* illustrates the reverse correlation method in the orientation domain (Ringach et al., 1997; Dragoi et al., 2002; Xing et al., 2005, 2011). The dynamic response of each site was smoothed with a rectangular window filter with a width of 20 ms (10 time points). We were then able to calculate the orientation tuning of each channel at different times relative to stimulus onset (see Fig. 1*B*). We used the stimulus-driven energy ratio (SER) to select visually driven sites. To define the SER, we calculated the energy of all orientations at different time delays as Energy(θ,t) = Resp(θ,t)^2^. We then averaged all orientations and defined the peak time as the time delay at which the energy reached its maximum. The SER was then calculated as the maximum energy divided by the mean energy before stimulus onset (−20 to 0 ms). SUAs and MUAs with SER >30 were used for further analysis.

##### Orientation tuning curves measured with drifting gratings

Orientation tuning curves were measured with drifting sinusoidal gratings in 24 probe placements (MUA; DD, with 15 probe placements and 148 sites; DY, with 6 probe placements and 66 sites; DQ, with 3 probe placements and 30 sites). Gratings were presented for 2 s, and response was the mean firing rate during this period. Orientation was varied over a range of 360° in steps of 15° or 20°. Each randomized orientation was presented at least 5 times (∼10 repeats in most cases). The size of the grating was 0.5°–2.5° in radius (at least 2 times larger than the RF of layer 4Cα), fixed within each recording session. The temporal frequencies of the drifting grating were 4.17 or 5. Other parameters (2 cycles/deg for spatial frequency and 90% for contrast) of the gratings were fixed in all sessions. Spontaneous firing rates were measured with a uniform screen of the same mean luminance as that of the grating stimuli.

##### Surround suppression measured with drifting gratings

After measuring the optimal orientation for each probe placement, we measured the size tuning by varying the radius of the stimulus patch from 0.015° to 6° for a sinusoidal grating (2 cycles/deg for spatial frequency and 90% for contrast) in 15 probe placements (MUA; DD, with 9 probe placements and 74 sites; DQ, with 4 probe placements and 43 sites; DK, with 2 probe placements and 24 sites). The temporal frequencies of the drifting grating were 4.17 or 5. The center of the stimulus was placed at the center of the RF. Each stimulus was presented for 2 s, with 10 repeats. The size tuning of each site was fitted with the difference of two Naka–Rushton functions (Naka and Rushton, 1966). Surround suppression was computed according to the following formula: surround suppression = [1 − (R_larg_/R_opt_)], where R_opt_ and R_larg_ denote the responses elicited by the optimal and largest radius stimuli, respectively. Thus, sites showing no suppression to large radius stimuli would have a surround suppression of 0, whereas those showing total response suppression would have a surround suppression of 1.

##### Laminar alignment

To align different probe placements in depth, we used the laminar pattern of MUA responses combined with current source density (CSD) analysis (Mitzdorf and Singer, 1979; Schroeder et al., 1998) of LFP signals. The MUAs and CSDs across laminar channels were measured during the presentation of random orientations. We averaged the responses in all stimulus conditions and calculated the MUA and CSD laminar patterns of every probe placement. We then summarized common signatures to guide laminar alignment. Because the thickness of the cortex and verticality of the probe differed between probe placements, we assigned the recording site of each channel to a relative depth (ReD) (Hawken et al., 1988). The ReD is the normalized cortical depth, ranging from 0 to 1. The boundaries between layers as a function of ReD were estimated based on previous anatomic (Lund, 1988; Callaway, 1998) and electrophysiological studies (Ringach et al., 2002; Yeh et al., 2009; Xing et al., 2012). Three signatures were used to calculate ReD. First, the CSD was smoothed in the cortical space. The location of the earliest current sink of CSDs (Cha_1_) was then referred to as the middle of layer 4Cα (Mitzdorf and Singer, 1979; Maier et al., 2011). We defined the ReD value of this signature (ReDs_1_) as 0.49. The response location with the earliest MUA responses was also calculated to define layer 4Cα for some probe placements (Maunsell and Gibson, 1992) that exhibited a blurry CSD pattern. For most probe placements, the location detected from MUA was the same as that for CSD. Second, half a channel above the uppermost channel (Cha_2_) exhibiting visually driven spiking responses (SER > 3.5, and its lower three continuous channels also met the condition of SER > 3.5) referred to as the boundary of cortex and pia mater was set as ReDs_2_ = 0). Third, the polarity inversion accompanied by the sink-source configuration (Cha_3_) was referred to as the boundary of layer 5 and 6 (ReDs_3_ = 0.81). This signature can be found in previous studies (Mitzdorf and Singer, 1979; Self et al., 2013), and was easy to detect in our data. After the three signatures were detected, we used the three signature pairs (Cha_1_ and Cha_2_; Cha_1_ and Cha_3_; Cha_2_ and Cha_3_) to calculate ReD between adjacent channels (ReD_inter_) as follows:

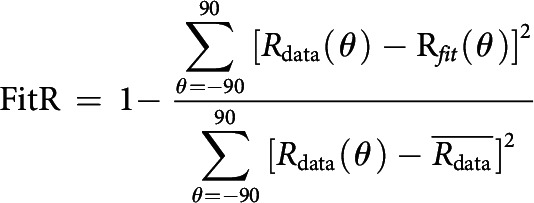


The ReD of every channel can be calculated from ReD_inter_ and one of the ReD values of the signatures as follows:

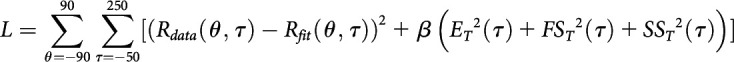


We then compared laminar patterns of MUA using the three ReDs calculated from different signature pairs, and selected the ReD with minimum difference from two standards. One is the difference between ReD of earliest response channel with the center of layer 4Cα (ReD = 0.49). The other was the difference between ReD of the latest response channel with center of layer 5 (ReD = 0.74). The criterion of selection was based on previous studies of the laminar pattern in macaque V1 (Maunsell and Gibson, 1992; Xing et al., 2012; van Kerkoerle et al., 2017).

##### Measure orientation selectivity

The orientation tuning curves were shifted so that their preferred orientation was 0° (see Fig. 1*B*). We then fitted tuning curves with the von Mises function (Khatri and Mardia, 1977) and used the fitted tuning curves (spaced from −90° to 90°, at 1° intervals) to estimate two aspects of orientation selectivity, as follows. We found the peak response (R_pref_) in the fitted curve; its orientation was defined as the preferred orientation. The responses to the orientation of 90° on either side of its preferred orientation were defined as the orthogonal response (R_orth_). We computed the ratio R_orth_/R_pref_, which was defined as the ratio of orthogonal responses and preferred responses (O/P ratio) (Gegenfurtner et al., 1996; Ringach et al., 2002). We also subtracted R_orth_ and found the points on both sides of the peak at which the responses were half of the peak response. Half of the distance between the two points was defined as the bandwidth (Campbell et al., 1968; Rose and Blakemore, 1974). The fitting goodness was quantified to select reliable sites for subsequent analysis. We defined fitting reliability (FitR) as 1 minus the ratio of fitting residual and total variation of the data as follows:

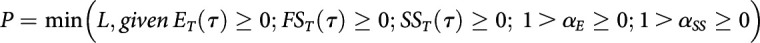
 Only sites with FitR >0.6 were used to analyze bandwidth.

##### Model fitting and evaluation

To dissect the excitation and two types of suppression that underlie orientation selectivity, we fitted a three-component model to the dynamic responses of each recorded channel; the experimental data contained 2718 data points (18 orientations × 151 time points). Parameters (α_E_, α_SS_, K_E_, K_SS_, θ_E_, θ_SS_, E_T_(τ), FS_T_(τ) and SS_T_(τ); τ from −50 to 250 ms, 2 ms interval) were searched to minimize L under constraints P by the MATLAB function “fmincon” as follows:

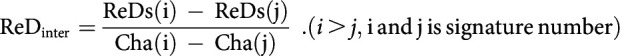


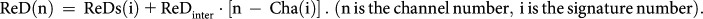


To evaluate the goodness of fit of the model, we defined the goodness of fit G described by the following equation:

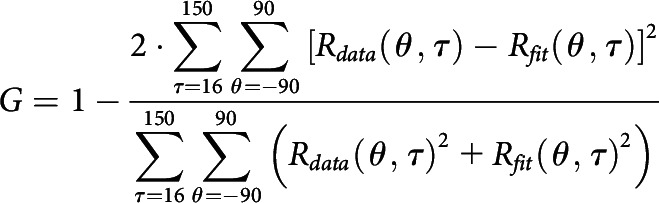


The fitting error in Figure 3 is described by the following:

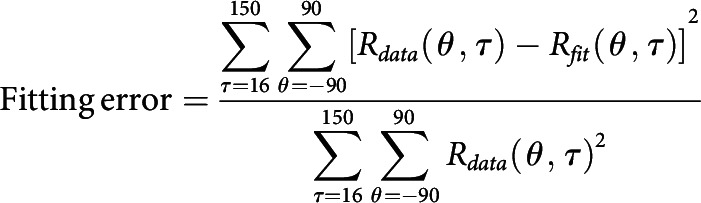


##### Statistics

All error bars and measures of dispersion represent mean ± SEM. All *p* values were two-tailed.

## Results

With a linear array (U-probe, 24 channels, 100 μm between adjacent channels), we simultaneously recorded the spiking activity and LFP evoked by grating patches presented at different orientations throughout the depth of V1 (Fig. 1*A*). SUA was isolated using offline spike sorting. We cross-correlated (also called reverse correlation, or spike-triggered average; see Materials and Methods) neural activity (SUA, MUA, and LFP) with stimulus orientations and calculated the dynamics of orientation tuning (Fig. 1*A*,*B*; see Materials and Methods). Based on the stimulus-driven MUA patterns (Fig. 1*C*) and the CSD patterns of visually evoked LFP (Fig. 1*D*), we defined the borders of adjacent cortical layers and aligned relative cortical depth for these channels (Materials and Methods). The MUA and CSD patterns for different probe placements are very similar, and they are similar to averaged MUA and CSD patterns, supporting the precise alignment of cortical depth and the assignment for cortical layers (Fig. 1*C*,*D*). Based on the aligned cortical depth of channels in each probe placement, we constructed the temporal development of orientation tuning.

### Temporal development of orientation selectivity and its laminar variation

**Figure 2.**
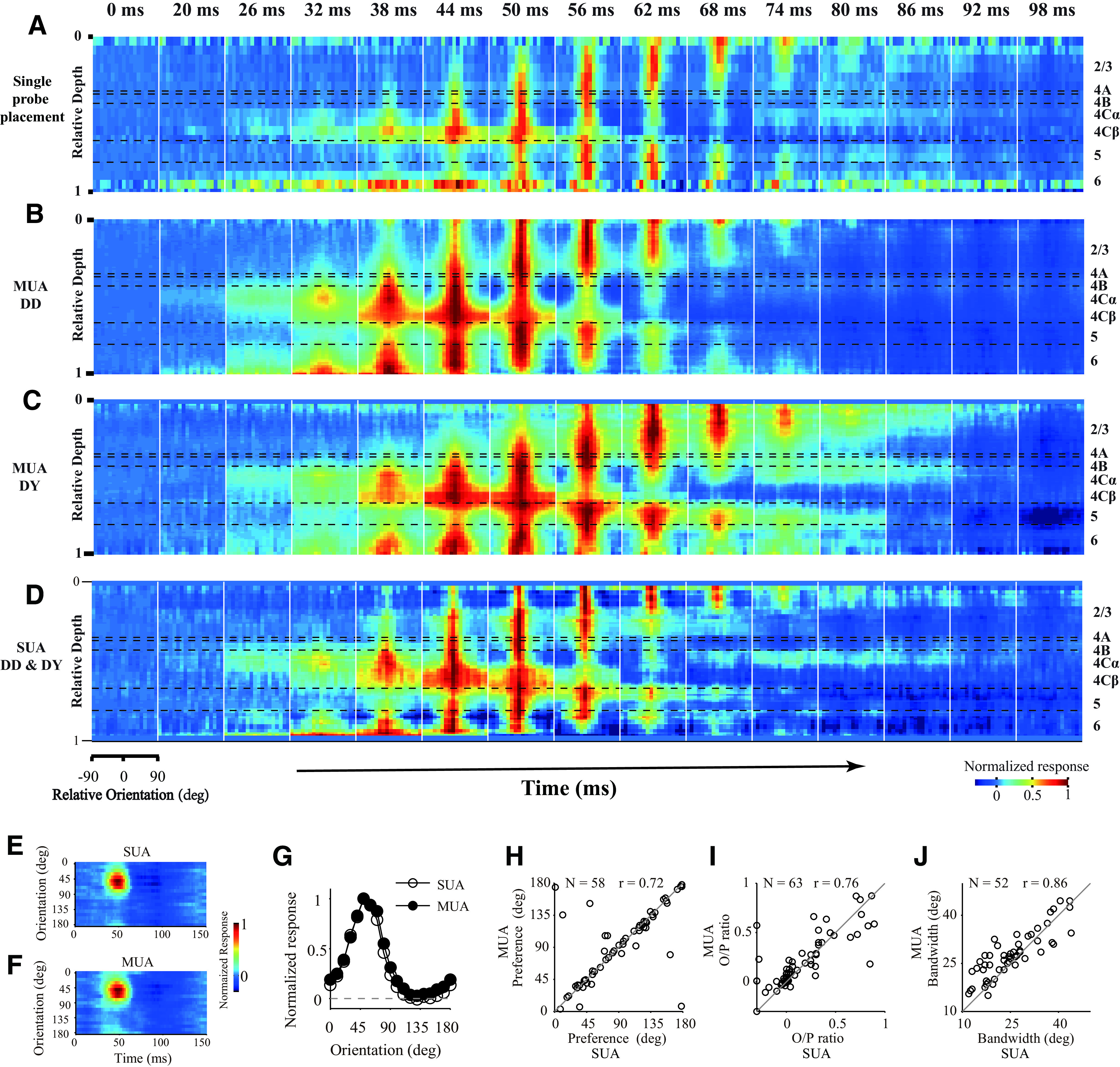
Population-averaged laminar pattern of orientation dynamics. ***A***, Laminar pattern of orientation dynamics in a single probe placement. The snapshots were plotted starting at 0 ms; then every 6 ms was selected from 20 to 98 ms after stimulus onset. Each snapshot shows orientation tuning at all depths within V1. MUA response strength was coded by color. Each site's response was normalized by its maximum value. The length of the sliding window for averaging across depth is 0.1 (relative depth). Horizontal black dashed lines indicate the laminar boundaries. ***B***, ***C***, Similar to ***A***, but averaged from multiple probe placements. ***B***, Averaged from 1 animal (DD, MUA; *N* = 293). ***C***, Averaged from another animal (DY, MUA; *N* = 114). ***D***, Averaged from 70 SUAs of 2 animals (DD, *N* = 58; DY, *N* = 12). Color scale applies to ***A–D***. ***E***, An example for orientation dynamics of single unit (SUA) from experiment DD2-u035-003, channel #5. ***F***, Orientation dynamics of MUA from the same recording site as in ***E***. ***G***, Orientation tuning of the example site (same as in ***E*** and ***F***). The tuning averaged from 36 to 58 ms shown in ***E*** and ***F***. Open circles represent SUA. Solid circles represent MUA. ***H***, The comparison of orientation preferences between SUA and MUA (*N* = 58, FitR > 0.45). Circular correlation coefficient (*r*) is 0.72. ***I***, The comparison of O/P ratio between SUA and MUA (*N* = 63, FitR > 0.1). Pearson's correlation coefficient (*r*) is 0.76. ***J***, The comparison of orientation bandwidth between SUA and MUA (*N* = 52, FitR > 0.6). Pearson's correlation coefficient (*r*) is 0.86 (for details of the measurement of O/P ratio and bandwidth, see Materials and Methods).

For a given probe placement, we shifted the preferred orientation of MUA responses from each channel to 0°, producing a spatiotemporal pattern for temporal development of orientation tuning across V1 layers (Fig. 2*A*). Such a spatiotemporal pattern shows a clear temporal order of response onset from input layers (L4C and L6) to output layers (L2/3, L4B, and L5) and distinct dynamics of orientation tuning clustered within each cortical layer. The temporal order of neural responses and laminar clustering of tuning dynamics for all probe placements were similar to each other, similar to the averaged laminar pattern of the orientation tuning, and consistent between 2 macaque monkeys (Fig. 2*B*,*C* for 2 Monkeys DD and DY), which again confirmed the depth alignment and laminar assignment. The dynamic response patterns for SUA (Fig. 2*D*) and MUA (Fig. 2*B*,*C*) were also similar to each other (Fig. 2*E-J* for individual SUA sites). To investigate the laminar pattern of orientation dynamics at a finer scale and with a better signal-to-noise ratio, we mainly analyzed MUA responses. Because the laminar patterns of MUA response dynamics were consistent between the 2 monkeys, all of the subsequent results are presented by combining data from the 2 monkeys (Monkey 1, DD, with 27 probe placements and 293 sites; Monkey 2, DY, with 9 probe placements and 114 sites).

**Figure 3.**
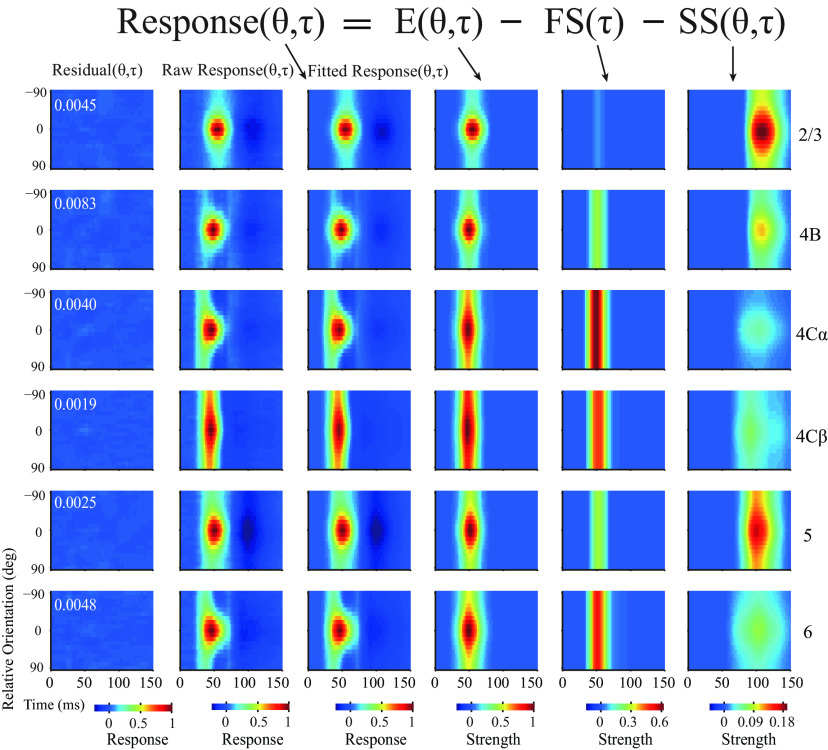
Three-component model for dynamic orientation tuning across V1 layers. Fitting population-averaged orientation dynamics within different layers used a three-component model. Different columns represent different aspects relative to model fitting. Different rows represent different layers. The second column represents raw normalized response (raw response). The third column represents the model fitted response pattern (fitted response). The first column represents the residual pattern (residual = raw response − fitted response). White numbers inset in the top left corner of each residual plot indicate the summed fitting error. The fourth to sixth columns represent three components (E, excitation; FS, fast suppression; SS, slow suppression) dissected from the dynamic response.

Two important dynamic features can be observed in the neural responses from single recorded sites (Figs. 1, 2) as well as in population-averaged responses (Figs. 1, 2, and the second column of Fig. 3). The first feature is that the response to the nonpreferred orientation (R_orth_) decays faster than the response of the preferred orientation (R_pref_). Moreover, the decay of R_orth_ can go below the baseline at ∼50-60 ms in input layers. The suppressive feature is also clear for individual Sites 12 and 14 shown in Figure 1*B* (also see blue regions at nonpreferred orientations in layer 4C and 6 at ∼50-60 ms in Fig. 2*A*,*D*). We can see that R_orth_ (blue dots) in the two example sites decays below the baseline from 54 to 64 ms. The other important feature for response dynamics is that R_pref_ peak negatively at ∼100 ms after stimulus onset (see the dark blue regions around preferred orientation in population responses of L2/3 and L5 in Fig. 2*C* and the second column of Fig. 3). The late suppression of R_pref_ is also clear for individual Site 6 in Figure 1*B* (red dots around 94 and 114 ms) which is in L2/3. The two features, early negative R_orth_ in L4C/6 and late negative R_pref_ in L2/3 and L5, strongly indicate that two suppressive mechanisms with different time course are involved in the neural responses in V1.

### Excitation and two types of suppression fully explain the V1 laminar response pattern

To distinguish excitation and the two suppressive mechanisms in different layers of Monkey V1, we modified a three-component model (Eqs. 1–6) (Xing et al., 2005, 2011) with one excitation and two types of suppression to fit the data from awake monkey.

In the three-component model (Eqs. 1–6), we assumed that the dynamic responses to stimulus orientations (R(θ,τ) in the second column of Fig. 3) were a linear combination of three components (Eq. 1): one excitatory component (E(θ,τ) in the fourth column of Fig. 3) and two suppressive components (a fast suppression component, FS(θ,τ), in the fifth column of Fig. 3 and a slow suppression component, SS(θ,τ), in the sixth column of Fig. 3). R, E, FS, and SS are all functions of stimulus orientations and time; and E, FS, and SS are assumed to be orientation and time separable, meaning that the function of orientation and time for E, FS, or SS can be simplified as the product of a function of time, X_T_(τ), and a function of orientation, V_X_(θ) (X = E, FS, or SS, in Eqs. 2–4). The orientation tuning of FS was flat, and the orientation tuning functions for E and SS were independent von Mises functions (Khatri and Mardia, 1977) plus an orientation-independent term α (Eqs. 5, 6). The orientation tuning functions V_E_(θ) and V_SS_(θ) are independent of each other.

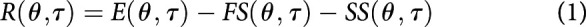


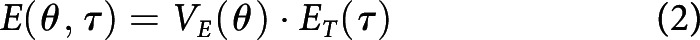


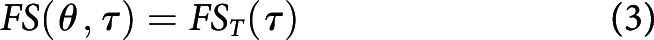


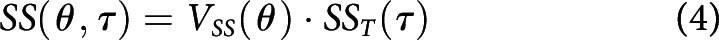


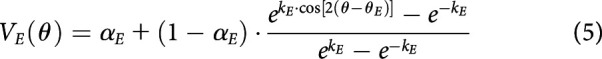


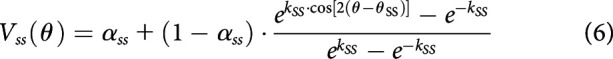


**Figure 4.**
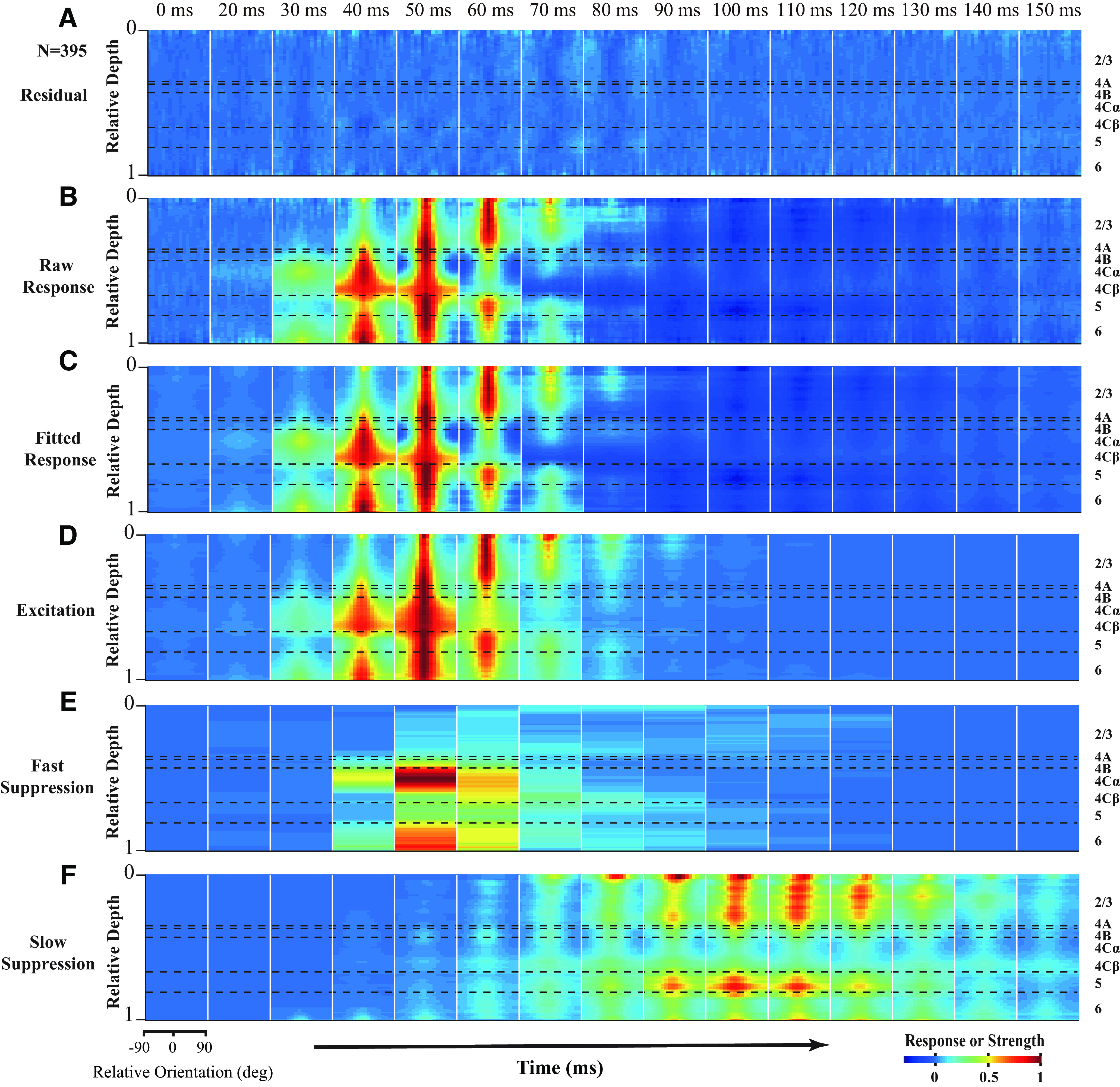
Laminar pattern of neural dynamics can be fully explained by one excitatory and two suppressive mechanisms. The snapshots are plotted starting at 0 ms; then every 10 ms was selected from 20 to 150 ms after stimulus onset. Each snapshot shows orientation tuning of components at different cortical depths. The length of the sliding window is 0.1 (relative depth) in cortical space. ***A–C***, The response at each depth was normalized by the corresponding peak value of the raw response at this depth. ***D–F***, The strength at each depth was normalized by the corresponding peak value of excitation at this depth. Patterns of FS (***E***) and SS (***F***) were further normalized by the maximum values of FS and SS, respectively. Horizontal black dashed lines indicate the laminar boundaries. Sites with fitting error <0.13 were used (*N* = 395).

The three-component model did a very good job to explain the dynamic responses to different stimulus orientations in different cortical layers both at the population-averaged level (Fig. 3, explained variance > 99%, fitting error < 1% for all layers) and at the level of individual sites (Fig. 4; for most MUA recording sites, 395 of 407, explained >86% of variance, with a rate of fitting error of <13%; mean ± SEM; goodness of fit, 0.947 ± 0.002, *N* = 407). These findings support the idea that V1 responses can be fully explained by one excitatory and two suppressive mechanisms.

To check whether the two suppressive mechanisms are necessary, we also tested an alternative hypothesis: V1 responses can be simply explained by an orientation-tuned excitation without any suppression, which is against the idea that V1 responses are mainly governed by an excitation and two types of suppression. We fitted the dynamic response with a feedforward model with only one excitation tuned to stimulus orientations. The goodness of fit for the feedforward model was significantly lower than that for the three-component model (mean ± SEM for goodness of fit; feedforward model, 0.778 ± 0.006; three-component model, 0.947 ± 0.002; *N* = 407, paired *t* test, *p* < 0.001). The proportion of sites with high goodness of fit (higher than 0.86) for the three-component model was much larger than the feedforward model (395 of 407, 97.1% for three-component model; 109 of 407, 26.8% for feedforward model). The good performance of our three-component model suggests that dynamic responses in all V1 layers were mainly governed by three neural mechanisms with distinct laminar distribution and neural dynamics, and probably different neural bases (Fig. 4).

### FS and SS have distinct laminar variations

**Figure 5.**
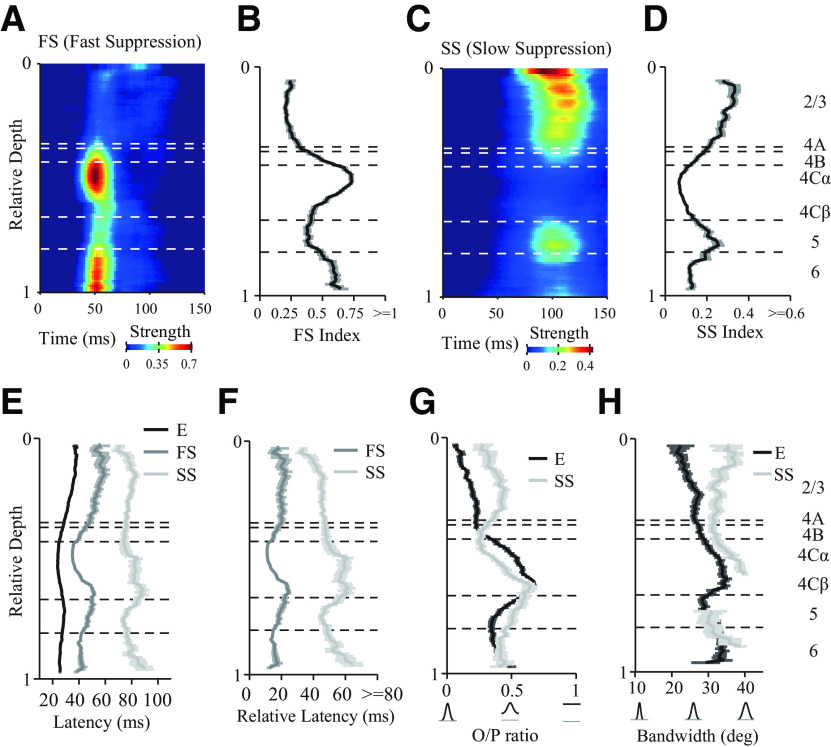
Laminar distribution of the strength, latency, and orientation selectivity for excitatory and suppressive components. ***A***, ***C***, Laminar patterns of mean strength for FS and SS. Mean strength averaged from all orientations and normalized by corresponding maximum value of excitation (E). The length of sliding window is 0.1 (in relative depth) in cortical space. ***B***, ***D***, Laminar distribution of FS and SS Index (MUA; *N* = 395). The index was defined as the maximum value of mean strength. ***E***, Latency of excitation (*N* = 395), FS (*N* = 346, FS Index > 0.12), and SS (*N* = 298, SS Index > 0.04). The latency was defined as the time at which each component first reached 2 × SDs of baseline fluctuations (−20 ms to 10 ms of raw dynamic response). ***F***, Latency difference between FS and excitation (latency of FS minus latency of excitation) and SS and excitation (latency of SS minus latency of excitation). ***G***, Laminar distribution of O/P ratio for excitation (*N* = 376, FitR > 0.1) and SS (*N* = 300, FitR > 0.1). ***H***, Laminar distribution of bandwidth for excitation (*N* = 297, FitR > 0.6) and SS (*N* = 102, FitR > 0.6). Some depths are not shown in the plot of SS because the sliding windows did not include more than two sites.

As shown in Figures 3 and 4, FS and SS exhibited marked differences in laminar distribution, dynamic properties, and orientation selectivity. FS was strongest in the input layers (L4Cα, L4Cβ, and L6; Fig. 5*A*,*B*; Table 1), whereas SS was strongest in the output layers (L2/3 and L5; Fig. 5*C*,*D*; Table 1). As their definitions suggest, FS was only slightly slower than excitation (Fig. 5*E*,*F*; Table 1), whereas SS was much slower than FS (Fig. 5*E*,*F*; Table 1). Interestingly, SS, on average, exhibited the shortest latency in the superficial layer close to layer 1 (Fig. 5*C*,*E*,*F*). SS in layer 2/3 and 5 was tuned to stimulus orientation but was weaker than the tuning of excitation in the same layers (Fig. 5*G*,*H*; mean ± SEM; L2/3, O/P ratio of excitation: 0.18 ± 0.02; SS: 0.40 ± 0.02; *N* = 99, paired *t* test, *p* < 0.0001; bandwidth of excitation: 23.45 ± 1.07 deg; SS: 32.86 ± 1.14 deg; *N* = 47, paired *t* test, *p* < 0.0001; L5, O/P ratio of excitation: 0.39 ± 0.03; SS: 0.52 ± 0.03; *N* = 49; paired *t* test, *p* < 0.0001; bandwidth of excitation: 30.77 ± 2.20 deg; SS: 30.14 ± 2.82 deg; *N* = 11; paired *t* test, *p* = 0.86; for details of the measurement of selectivity, see Materials and Methods).

**Table 1.** Detailed information for the strength and relative latency of FS and SS in different layers^a^

	FS index		SS index		Relative latency of FS		Relative latency of SS	
	Mean ± SEM	No. of sites	Mean ± SEM	No. of sites	Mean ± SEM (ms)	No. of sites	Mean ± SEM (ms)	No. of sites
L2/3	0.23±0.02	115	0.28±0.01	115	20.51±1.66	82	44.70±1.48	112
L4B	0.46±0.04	29	0.17±0.02	29	18.07±3.00	27	46.32±3.62	25
L4Cα	0.72±0.02	68	0.07±0.01	68	11.35±0.44	68	60.32±6.05	31
L4Cβ	0.47±0.02	57	0.10±0.01	57	21.31±1.30	55	61.03±5.10	31
L5	0.39±0.03	63	0.23±0.02	63	18.25±1.64	55	46.81±2.18	54
L6	0.59±0.03	52	0.12±0.01	52	14.94±1.25	51	58.82±5.73	34

*^a^*The FS and SS index was defined as the maximum value of mean strength. The latency was defined as the time at which each component first reached 2 × SDs of baseline fluctuations (−20 ms to 10 ms of raw dynamic response). Relative latency was defined as the latency difference between FS and excitation (latency of FS minus latency of excitation) and SS and excitation (latency of SS minus latency of excitation).

**Figure 6.**
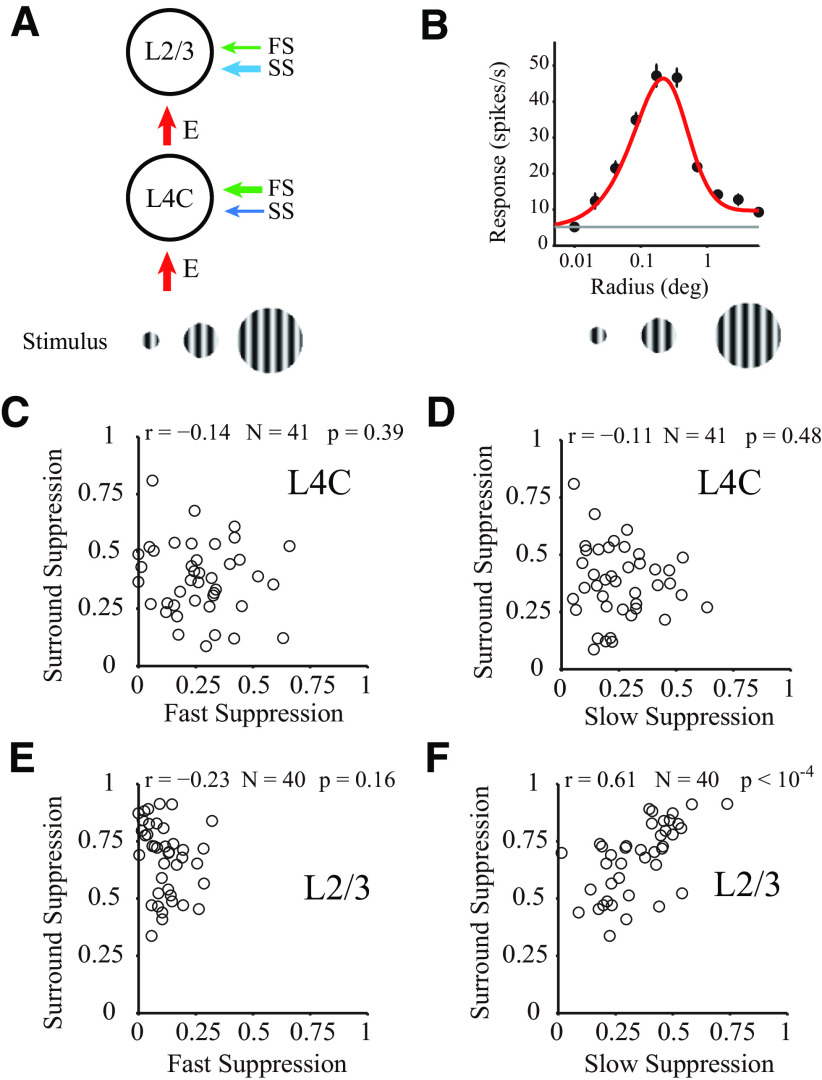
Correlation of two types of suppression with surround suppression. ***A***, Schematic of the cascading relationship between the input layer (L4C) and output layer (L2/3). Stimuli were drifting sinusoidal gratings with different radius. Arrow thickness represents the strength of two types of suppression and excitation. ***B***, Examples of individual tuning curves measured with drifting gratings of varying radius. Red curves indicate fits to the data (black dots) using the difference between two Naka–Rushton functions. Gray line indicates the spontaneous rate of firing. The example site was located in layer 2/3. ***C***, ***D***, Relationship between surround suppression and two types of suppression (***C***, FS; ***D***, SS). Scatter plot for all sites of layer 4C. Strength of suppression defined as averaged strength from 0 to 200 ms. ***E***, ***F***, Similar to ***C***, ***D*** but for all sites of layer 2/3.

The results described in this section show that laminar processing in V1 can be simplified into two stages, which included an input layers (L4C) stage with strong FS and an output layers (L2/3) stage with strong SS (Fig. 6*A*). The segregated distribution of two types of suppression raise a key question: what is the function of the two distinct types of suppression for laminar processing?

### SS is related to surround suppression in V1 output layers

One of the canonical cortical functions in V1 is surround suppression, indicating the ability of V1 to integrate spatial context (Allman et al., 1985). V1 neurons respond best to stimuli of optimal size falling on the cell's RF, and are suppressed by stimuli larger than the optimal stimulus size. To elucidate the relationship between the two suppression types and surround suppression, patches of drifting gratings of increasing size centered over the RF (Fig. 6*A*) were used in 15 probe placements to estimate surround suppression (for details, see Materials and Methods). For most recording sites in V1, there was surround suppression when stimulus size changed from the optimal size to a larger size (for an example site, see Fig. 6*B*).

Interestingly, only SS in the output layer was strongly positively correlated with surround suppression (L2/3; Fig. 6*F*; *r* = 0.61, *p* < 10^−4^, *N* = 40). The correlation between FS and surround suppression in the output layer was weak and not significant (Fig. 6*E*; *r* = −0.23, *p* = 0.16, *N* = 40). Neither FS nor SS correlated with surround suppression in input layer (L4C; *N* = 41; Fig. 6*C* for FS and surround suppression, *r* = −0.14, *p* = 0.39; Fig. 6*D* for SS and surround suppression, *r* = −0.11, *p* = 0.48). The significant positive correlation between surround suppression (measured using a drifting grating) and SS (estimated using a flashed grating) suggests that SS, but not FS, may participate in spatial context processing and caused the enhancement of surround suppression in laminar processing in the output layer of V1.

To be noticed, there is also moderate surround suppression in input layers measured with drifting gratings (Fig. 6*C*,*D*) (Solomon et al., 2002; Alitto and Usrey, 2008; Henry et al., 2013). However, we do not see significant correlation between the weak SS and the moderate surround suppression in input layers. It is possible that the weak SS in the input layers also represents surround mechanism participating in spatial context processing, but the surround activated by rapidly presented stimuli was weaker than the surround activated by drifting gratings; therefore, we do not see any correlation between surround suppression and the SS in V1 input layer.

### FS improves orientation selectivity in V1 input layers

We next determined how the two suppression types participate in orientation processing. Several previous studies reported that broadly tuned suppression plays a major role in the enhancement of orientation selectivity (Sillito, 1975; Ringach et al., 2003; Liu et al., 2011; Xing et al., 2011). However, other studies found that inhibitory tuning is as narrow as excitatory tuning, and concluded that inhibition cannot sharpen orientation tuning (Ferster, 1986; Anderson et al., 2000; Tan et al., 2011). The current results revealed that FS and SS in macaque V1 were both broadly tuned, indicating a possible contribution to orientation selectivity. The functional differences between the two types of suppression in orientation processing and the ways in which they modulated orientation information in different layers are considered in more depth below.

**Figure 7.**
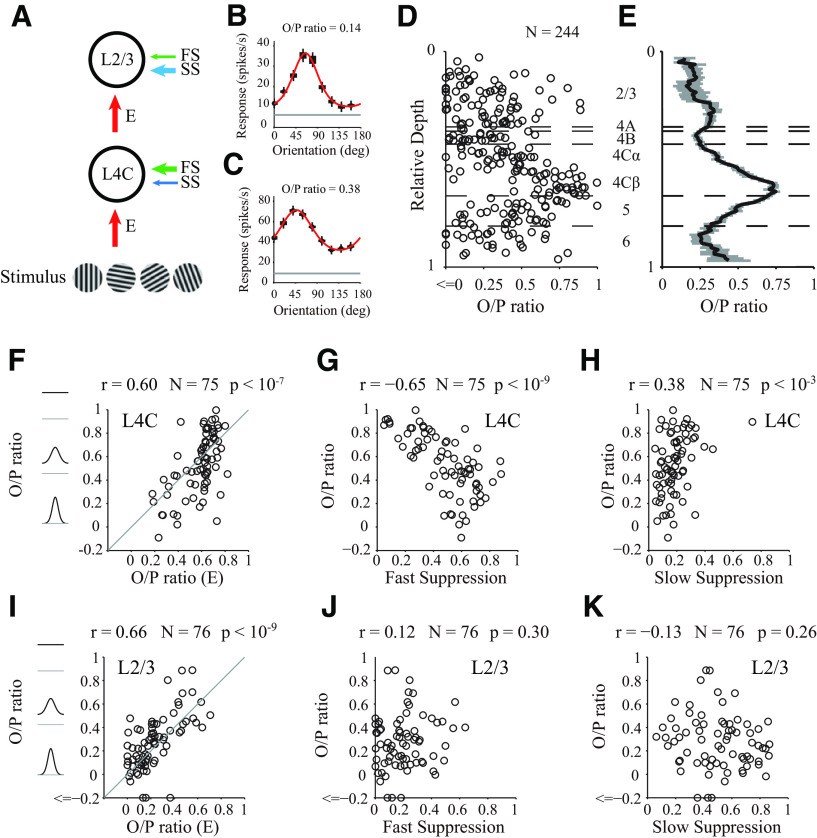
Correlation of three components with orientation selectivity. ***A***, Schematic of the cascade relationship between input layer (L4C) and output layer (L2/3). Stimuli were drifting sinusoidal gratings with different orientations. Arrow thickness represents the strengths of suppression and excitation. ***B***, ***C***, Examples of individual tuning curves measured by drifting gratings of varying orientation. Red curves indicate fits to the data (black dots) using the von Mises function. Gray line indicates the spontaneous rate of firing. Example site in ***B*** located in layer 2/3. Example site in ***C*** located in layer 4C. ***D***, Scatter plot of O/P ratio, measured with drifting gratings, against relative depth (*N* = 244). Horizontal black dashed lines indicate the laminar boundaries. ***E***, Running average of O/P ratio at different cortical depth in ***D***. The length of the sliding window for averaging across depth is 0.1 (relative depth) in cortical space. ***F–H***, Relationship between O/P ratio calculated from tuning curves measured by drifting gratings and different mechanisms dissected from dynamic response (***D***, O/P ratio of excitation; ***E***, FS; ***F***, SS). Scatter plot for all sites of layer 4C. Strength of suppression defined as averaged strength from 0 to 200 ms. ***I–K***, Similar to ***F–H***, but for all sites of layer 2/3.

To determine how orientation selectivity relates to inhibitory and excitatory mechanisms, we also measured orientation tuning curves with drifting sinusoidal gratings as stimuli, with 24 probe placements (for details, see Materials and Methods; Fig. 7*A*), as shown for two example MUA sites in Figure 7*B*, *C*. The orientation selectivity was defined as the O/P ratio (see Materials and Methods; for all individual sites and laminar distribution, see Fig. 7*D*,*E*). The O/P ratio of excitation (measured using a flashed grating stimulus) was positively correlated with the O/P ratio (measured by drifting grating, mean ± SEM of O/P ratio; for L2/3, 0.255 ± 0.037, *N* = 76; for L4B, 0.228 ± 0.033, *N* = 22; for L4Cα, 0.356 ± 0.032, *N* = 35; for L4Cβ, 0.689 ± 0.032, *N* = 40; for L5, 0.464 ± 0.035, *N* = 40; for L6, 0.288 ± 0.057, *N* = 29) in both input layers (Fig. 7*F*; L4C; *r* = 0.60, *p* < 10^−7^, *N* = 75) and the output layer (Fig. 7*I*; L2/3; *r* = 0.66, *p* < 10^−9^, *N* = 76). However, in the input layer, the O/P ratio was also significantly negatively correlated with the strength of FS (Fig. 7*G*; L4C; *r* = −0.65, *p* < 10^−9^, *N* = 75). In the output layer, FS and the O/P ratio were not significantly correlated (Fig. 7*J*; L2/3; *r* = 0.12, *p* = 0.30, *N* = 76). In contrast to FS, the correlation between SS and the O/P ratio was weak in the input layers (Fig. 7*H* for L4C, *r* = 0.38, *p* < 10^−3^, *N* = 75) and not significant in the output layers (Fig. 7*K* for L2/3, *r* = −0.13, *p* = 0.26, *N* = 76). Overall, we found that FS made a laminar-specific contribution to orientation selectivity. Our results are consistent with the notion that FS enhances orientation selectivity at the input layers of V1 by reducing neural responses to orthogonal orientation.

### The output layer inherits the effects of FS from the input layer

Different from results in the input layer (L4C), orientation selectivity in the output layer (L2/3) was only significantly correlated with the selectivity of excitation (Fig. 7*I-K*), indicating that excitation but not suppression played a major role in L2/3. Although it is currently unclear how excitation in L2/3 is generated, several sources could be involved, including feedforward excitation from L4C, recurrent excitation within L2/3, and recurrent excitation between L2/3 and L5 (Lund, 1988; Callaway, 1998). Because of the diversity of the sources of excitation, whether the selectivity of L2/3 can directly benefit from FS generated in L4C remains an open question. If feedforward excitation plays an important role, FS in L4C can indirectly enhance orientation selectivity in L2/3, and there will be a correlation between the strength of FS in L4C and selectivity in L2/3. In contrast, if recurrent excitation is the dominant determinant of selectivity in L2/3, FS in L4C and orientation selectivity of L2/3 would be expected to be uncorrelated.

**Figure 8.**
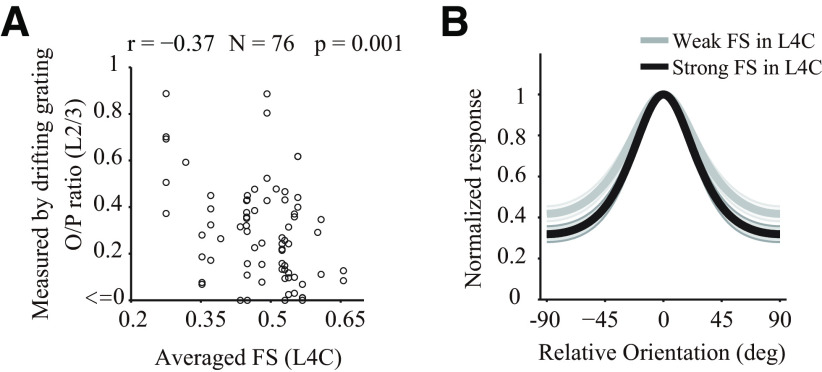
Cascade effects of FS across layers. ***A***, Scatter plot of FS of L4C and O/P ratio of L2/3 measured by drifting grating. For each MUA site of L2/3 (*N* = 76), we calculated the averaged integrated FS strength (from 0 to 200 ms) of all simultaneously recorded L4C sites. The Pearson's correlation coefficient (*r*) is −0.37. ***B***, Population-averaged orientation tuning curves of L2/3 sites (*N* = 30 for each population). Black line indicates the average for the strong FS population in layer 4C (top 30). Gray line indicates the average for the weak FS population in layer 4C (last 30).

To further investigate the functional implications of our findings, we investigated the relationship between FS of L4C and orientation selectivity of L2/3. For each site of L2/3 (*N* = 76), we averaged FS of all simultaneously recorded L4C sites. The O/P ratio of L2/3 exhibited a significant negative correlation with the averaged FS of L4C (Fig. 8*A*; *r* = −0.37, *p* = 0.001, *N* = 76). To further examine this effect, we separated L2/3 sites into weak and strong populations based on the simultaneously measured strength of FS in L4C (*N* = 30 for each population). A strong FS population exhibited sharpened orientation tuning with reduced orthogonal response compared with a weak FS population (Fig. 8*B*). These results indicate that the effects of FS in the input layer can be directly inherited by the output layer through feedforward circuitry, and that these inherited suppressive effects can shape selectivity of output layer.

### Laminar variation of orientation preference

In addition to the neural mechanisms for variations of orientation selectivity across V1 layers, several studies (Bauer et al., 1980, 1983; Bauer and Dow, 1989) have reported that preferred orientations at different layers within a column perpendicular to V1 surface can be substantially different, especially between upper/middle layers (L2/3/4C) and lower layers (L5/6). We also checked whether the shift of preferred orientation between layer 4C and layer 5/6 are different from the orientation shift between layer 4C and layer 2/3 in our dataset.

**Figure 9.**
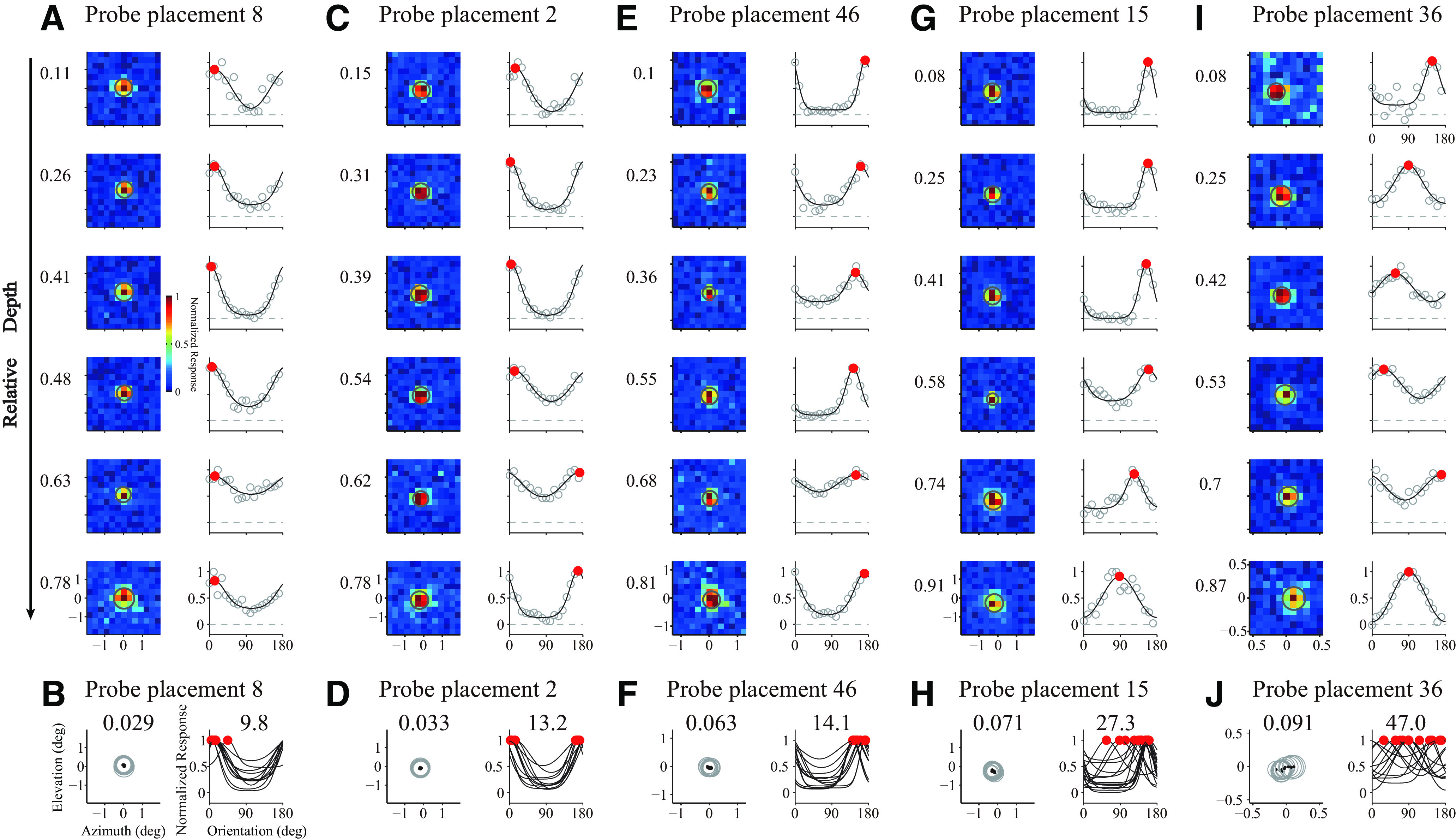
RF mapping and preferred orientation estimation. ***A***, RF mapping and preferred orientation estimation for an example probe placement. Left columns represent RFs as heat maps for sites through the depth of V1. The relative depth was labeled left to heat maps. Gray circle in each heat map represents the RF estimated by fitted Gaussian functions. Each RF map was normalized by its maximum value. Right columns represent orientation tunings of the same sites. Black curves indicate model (von Mises function) fits to the data (gray dots). Filled red dots represent sites' preferred orientations. ***B***, RFs and orientation tunings of all sites recorded in the example probe placement in ***A***. Only sites with well-fitted orientation tuning and RF are shown. Black dots represent RF centers of V1 sites. Left, The mean value of center distances among all pairs of RFs within V1 (MCD, 0.029) for the probe placement. Right, The mean value of absolute difference of orientation preferences among all pairs of tunings within V1 (MPD, 9.8) for the probe placement. ***C–J***, Similar to ***A*** and ***B***, but for another 4 probe placements. Color scale applies to all heat maps.

**Figure 10.**
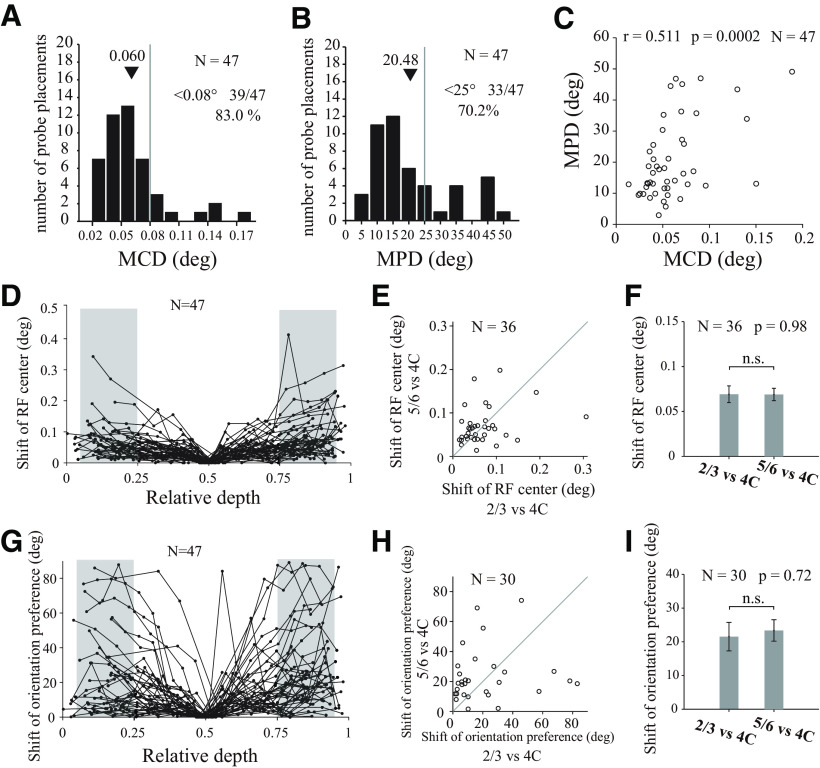
Shifts of RF centers and orientation preferences. ***A***, Distribution for MCDs from individual probe placement (*N* = 47 sessions). Black triangles represent mean value of the MCDs in all sessions. Gray dashed line indicates the threshold (0.08°). MCDs <0.08 visual angle occupy 83.0% (39 of 47) sessions. ***B***, Distribution for mean value of absolute differences of orientation preferences (MPD) from individual probe placement (*N* = 47 sessions). Gray dashed line indicates the threshold (25°). MPDs <25°occupy 70.2% (33 of 47) sessions. ***C***, Relationship between MCDs and MPDs for all valid probe placements. ***D***, Shift of RF centers against relative depth. The shift of RF center of each recording site was defined as the center distance between the site's RF center and the RF center of L4C in the same probe placement (the site nearest to relative depth of 0.5). Each dotted line indicates 1 probe placement. Shaded areas represent regions in L2/3 or L5/6 (located at L2/3 and L5/6; range of relative depth, 0.05-0.25 for L2/3, 0.75-0.95 for L5/6) for further analysis in ***E*** and ***F***. ***E***, Scatter plot for shift of RF centers in L2/3 (relative to L4C in the same probe placement) against shift of RF center in L5/6 (relative to L4C in the same probe placement). Probe placements (*N* = 36) were included if L2/3, L4C, and L5/6 all have valid recording sites. ***F***, Average shift of RF centers in L2/3 and L5/6 (both are relative to L4C; paired *t* test; n.s., not significant). ***G***–***I***, Similar to ***D***–***F***, but for shift of orientation preferences.

For each probe placement, we estimated both the preferred orientation and the center position of the RF for each recorded site (see examples in Fig. 9). Most of our probe placements are perpendicular to V1 surface, according to the mean distance of all pairs of RF centers in each probe placement (MCD). The mean value of MCDs, 0.06 degree in visual angle (Fig. 10*A*,*D* for individual sites), is much smaller than the mean size of the V1 RFs (0.3° in diameter). There is no significant difference (Fig. 10*E*,*F*; paired *t* test, *N* = 36, *p* = 0.98) between mean shift of RF centers in layer 2/3 and those in layer 5/6 (both relative to RF centers in layer 4C).

**Figure 11.**
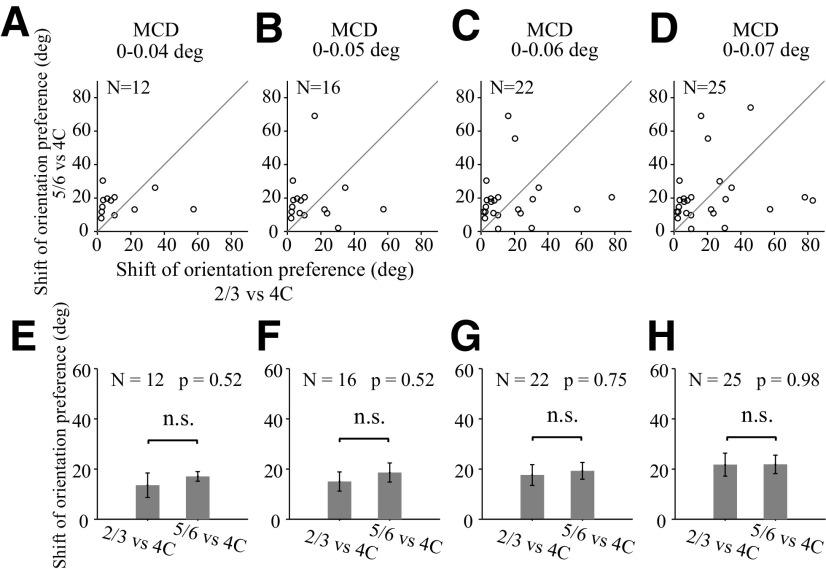
Comparison of the shifts of orientation preferences between upper and lower layers relative to the input layers. Each column represents the comparison of shifts of orientation preferences between upper (L2/3) and lower (L5/6) layers relative to the input layers (L4C), with different selection criteria for perpendicular probe placements. The selection criteria are based on MCDs from individual probe placement. Probe placement with MCDs <0.04 deg for ***A*** and ***E***, MCDs <0.05 deg for ***B*** and ***F***, MCDs <0.06 deg for ***C*** and ***G***, and MCDs <0.07 deg for ***D*** and ***H***. ***A–D***, Scatter plots for shift of orientation preferences in L2/3 against those in L5/6 (relative to orientation preferences in L4C). Probe placements with valid recording sites in both L2/3 and L5/6 were used. ***E–H***, Average values of shifts of orientation preferences in L2/3 and average values of those L5/6. Nonsignificant values (paired *t* test; n.s., not significant).

We analyzed orientation preferences in the way similar for the analysis on RF centers. The mean difference of orientation preference (MPD) is 20.48° (Fig. 10*B*). Orientation preference shifts is significant correlated with RF center shifts (Fig. 10*C*; *r* = 0.511, *N* = 47, *p* = 0.0002). We used RF center shifts (Fig. 10*A*) as a criterion to select perpendicular probe placements. There are shifts of orientation preferences for layer 2/3 and layer 5/6 relative to those in layer 4C (Fig. 10*G*). However, for probe placements perpendicular to V1 surface, judged by RF center shift (Fig. 10*A*; center shifts are <0.08°), the shift of orientation preferences in layer 2/3 and layer 5/6 is not significantly different (Fig. 10*H*,*I*; mean ± SEM; for L2/3 relative to L4C, 21.53 ± 4.22 deg; for L5/6 relative to L4C, 23.36 ± 3.18 deg; paired *t* test, *N* = 30, *p* = 0.72). We further compared the shift of orientation preferences in L2/3 and those in L5/6 with stricter criteria for selecting perfectly perpendicular probe placements (MCD is <0.04°, 0.05°, 0.06°, and 0.07°), and we did not find significant differences in any condition (Fig. 11). Our results are consistent with the notion that orientation preferences change in a smooth fashion within V1 column.

## Discussion

**Figure 12.**
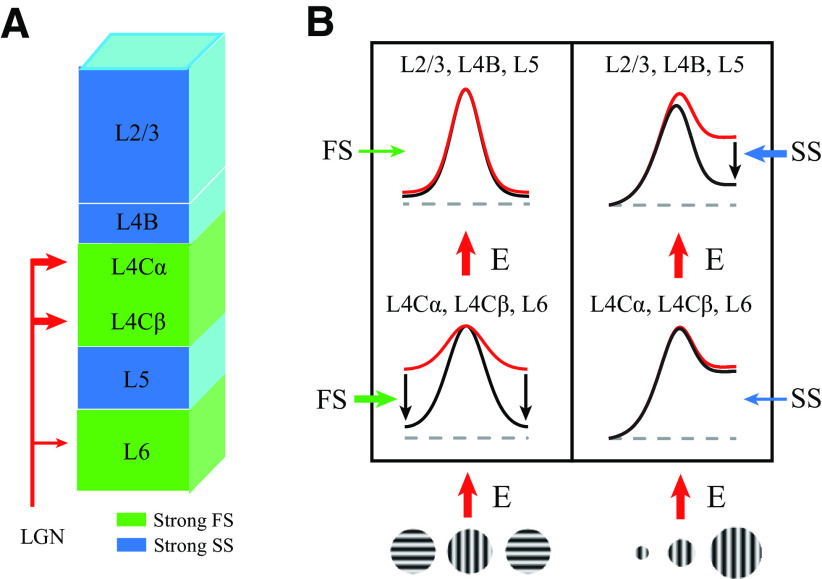
Summary of different types of suppression for laminar processing. ***A***, Schematics of the results showing the distribution of FS and SS in V1. Green shading represents input layers (L4Cα, L4Cβ and L6) that receive geniculocortical input and have strong FS. Blue shading represents output layers (L2/3, L4B, and L5) which have strong SS. ***B***, Schematics of the results regarding the functional properties of two types of suppression in orientation and spatial context processing. Excitation (red arrows) is the initial input of each layer. Red curve indicates tuning inherited from excitation. Black curve indicates the tuning modulated by suppression. The thickness of the arrows of FS (green arrows) and SS (blue arrows) represents the strength. The arrows around each tuning curve indicate the change in response magnitude caused by suppression.

Our experimental results provide a complete picture of the temporal dynamics of orientation selectivity across macaque V1 layers (Fig. 2). Based on the temporal dynamics of orientation selectivity, we distinguished one excitatory and two suppressive components that collaboratively process visual information across V1 layers (Figs. 3, 4). The two suppressive components, FS and SS, exhibited distinct laminar distributions and caused diversity of neural dynamic responses to stimulus orientations (Figs. 5, 12*A*). We further investigated how suppressive mechanisms contribute to orientation and spatial context processing by laminar circuitry (summarized in Fig. 12*B*). Laminar processing in V1 can be simplified into two stages (input layers and output layers). FS largely modulated tuned excitation by reducing the excitation for nonpreferred orientations in the input layer, whereas SS contributed to integrating spatial context and was correlated with surround suppression in the output layer. Together, these results suggest that two suppressive mechanisms have distinct laminar distributions and play different functional roles in macaque V1.

### FS and SS in V1 layers

Based on the laminar distributions and time courses of fast and SS, we believe that these two types of suppression arise from different neural circuitries. FS is likely to be a local process because it is only slightly slower than excitation, with a 10 ms time delay (Figs. 4, 5*E*,*F*) and is located in layers with more local connections. SS is likely to be because of long-range connections or feedback because it is substantially slower than FS (∼40 ms time delay compared with excitation and FS; Fig. 5*E*,*F*) and is mainly located in the output layers, which contain large numbers of unique horizontal (Stettler et al., 2002; Lund et al., 2003) and feedback connections (Rockland and Pandya, 1979; Stettler et al., 2002). Interestingly, the earliest SS appeared in the superficial layer close to layer 1 (Fig. 5*C*,*E*,*F*). Our results regarding SS, based on spike activity, were highly similar to those reported in a recent study (Bijanzadeh et al., 2018), based on LFP and CSD, which suggested that SS could be because of feedback connections. In addition, the two types of suppression may be governed by different neurotransmitter receptors. The earlier latency and shorter duration of FS indicate that the suppression is related to GABA_A_ receptors with fast and transient synaptic function (Marienhagen et al., 1997). GABA_B_, with slow and sustained synaptic properties (Marienhagen et al., 1997), may contribute to SS. Coincidentally, the laminar distribution of SS in our results is similar to the laminar distribution of GABA_B_ density in V1 (Eickhoff et al., 2007).

### Neural mechanisms for orientation selectivity

Orientation selectivity is an important function in V1 for understanding cortical computational principles (Hubel and Wiesel, 1968; Priebe and Ferster, 2012). Several neural mechanisms, including the organization of excitatory LGN input (Hubel and Wiesel, 1962; Reid and Alonso, 1995), cortical inhibition, nonlinear transduction of membrane potential to spiking activity (Gardner et al., 1999; Tan et al., 2011), and recurrent excitation (Somers et al., 1995; Hansel and Sompolinsky, 1996) have been proposed to enhance orientation selectivity. However, previous theories related to these neural mechanisms have been controversial and mutually exclusive. A recent study used a computational model to distinguish three mechanisms (excitation, inhibition, and nonlinearity), supporting the notion that excitatory input is the most important mechanism contributing to selectivity and its variation in V1 (Goris et al., 2015). Our results revealed that, in addition to excitation, inhibition also plays important roles and neural mechanisms for orientation selectivity might be layer-specific. The relationships between inhibitory effects (FS) and orientation selectivity differ between input and output layers (Fig. 7), suggesting that there are layer-specific mechanisms for orientation selectivity in V1. In input layers (L4C), both FS and excitation relate to selectivity (Fig. 7*F*,*G*); but in output layers (L2/3), only excitation relates to selectivity (Fig. 7*I*,*J*). More importantly, although orientation selectivity of L2/3 is seemingly only governed by excitation, we found that FS in L4C was also significantly correlated with selectivity of L2/3. The effects of FS in L4C can be inherited by L2/3 and contribute to selectivity of L2/3.

### Laminar variation of orientation selectivity

In addition to the overall variation of orientation selectivity for SUA and MUA in V1, differences of mean orientation selectivity across V1 layers (laminar variation of orientation selectivity) were reported by studies in various species, including macaque monkey (Livingstone and Hubel, 1984; Ringach et al., 2002; Gur et al., 2005), cat (Martinez et al., 2002), mouse (Niell and Stryker, 2008), rat (Girman et al., 1999), and tree shrew (Chisum et al., 2003). The mechanisms underlying this laminar variation remain unclear (Hirsch and Martinez, 2006). A previous study by Martinez et al. (2002) found that the relative tuning of excitation and inhibition changed with laminar variation, providing a mechanistic view of laminar variation in cat V1. Given the fundamental differences in the neuronal mechanisms underlying orientation selectivity between species (Bosking et al., 1997; Ohki et al., 2005; Hirsch and Martinez, 2006; Scholl et al., 2013), it is still important to understand the mechanisms underlying laminar variation of orientation selectivity in macaque V1.

We also found that mean value of O/P ratio in each V1 layer varies (Fig. 7*E*). Interestingly, the significant differences of O/P ratio among V1 layers are mostly from comparisons between L4Cβ, a sublayer of L4C, and other V1 layers. The O/P ratio in L4Cβ is significantly larger than all other layers (one-way ANOVA test, *p* < 0.01 for difference between L4Cβ and all other layers). Surprisingly, the O/P ratio in L4Cα, the upper half of L4C next to L4Cβ, is only significantly lower than that in L4Cβ, but not different from that in any other layer (one-way ANOVA test, *p* < 0.01 for the difference between L4Cα and L4Cβ; *p* > 0.05 for difference between L4Cα and all other layers except L4Cβ).

Our finding is consistent with previous studies in macaque monkey (Ringach et al., 2002; Gur et al., 2005), but it is very contrary to the general impression that orientation selectivity of input layer (L4C) is low or nonexistent in macaque V1 and orientation selectivity in L2/3 is much better than that in L4C. We think the results can be explained by the laminar variation of excitation and suppression in V1 layers revealed by the current study. First, the L4Cα and L4Cβ substantially differ for their O/P ratio, because FS in L4Cα is stronger than that in L4Cβ (Fig. 5*A*,*B*; mean ± SEM; FS for L4Cα, 0.72 ± 0.02, *N* = 68; FS for L4Cβ, 0.47 ± 0.02, *N* = 57; two-sided *t* test, *p* < 10^−8^), and the O/P ratio of excitation in L4Cα is lower than that in L4Cβ (Fig. 5*G*,*H*, black curve; two-sided *t* test, *p* < 10^−7^), although excitation in both L4Cα and L4Cβ is poorly tuned to orientation (Fig. 5*G*, black curve; mean ± SEM; for L4Cα, 0.474 ± 0.025, *N* = 65; for L4Cβ, 0.656 ± 0.010, *N* = 46). It is the combination of excitation and FS that leads to a large difference for O/P ratio between L4Cα and L4Cβ. Second, the comparable O/P ratio in L2/3 and L4Cα is because of the following two factors. (1) FS in L2/3 is generally weak (mean ± SEM; for L2/3, 0.23 ± 0.02, *N* = 115; one-way ANOVA test, *p* < 10^−5^ for comparison between L2/3 and all other layers; see Table 1). (2) Under our experimental conditions, excitatory inputs to L2/3 are related to neural activity in L4Cα more than L4Cβ because achromatic (black/white) stimuli used in our experiment activated L4Cα more than L4Cβ (mean ± SEM; firing rates for L4Cα, 70.3 ± 4.5 spikes/s, *N* = 68; for firing rates for L4Cβ, 39.9 ± 2.6 spikes/s, *N* = 59; two-sided *t* test, *p* < 10^−6^). The weak FS and L4Cα-dominant excitation in L2/3 lead to a comparable O/P ratio in L2/3 and L4Cα. In summary, we think that the laminar variation of the O/P ratio is largely because of the laminar-specific excitation and FS in V1, which starts in V1 input layer.

Our explanation for the laminar variation of orientation selectivity measured by O/P ratio does not indicate that no other mechanism is involved in processing orientation information in L2/3. There is a significant difference between L2/3 and L4Cα for their tuning bandwidth, another way to measure orientation selectivity (mean ± SEM for bandwidth; 31.17 ± 0.92 deg, *N* = 69, for L2/3; 30.61 ± 1.15 deg, *N* = 22, for L4B; 35.91 ± 1.03 deg, *N* = 35, for L4Cα; 41.74 ± 0.84 deg, *N* = 25, for L4Cβ; 37.86 ± 1.13 deg, *N* = 36, for L5; 37.28 ± 1.23 deg, *N* = 25, for L6; one-way ANOVA test, *p* < 0.01 for comparison between L2/3 and all other layers except L4B). The significant change of orientation bandwidth between L2/3 and L4Cα indicates that recurrent connections within L2/3, interlaminar connections between L2/3 and L5, or static nonlinearity in L2/3 may also contribute to enhance orientation selectivity in layers 2/3.

### General computation across V1 layers

The laminar-specific excitation and two types of suppression found in our study may represent a general computation for information processing of other visual features, including spatial frequency, brightness, and color. Strong and FS in input layers can increase global feature selectivity (Xing et al., 2011), by suppressing thalamocortical inputs responding to nonpreferred features. The output layers receive multiple sources of forward and recurrent excitatory connections. SS in output layers can play important roles in information integration, feedback control, and feature binding.

From the laminar distributions of the excitation and two types of suppression in the current study, we can summarize laminar processing into two distinct subnetworks. The first subnetwork operates at the thalamocortical stage within input layers, which transforms thalamic input and generates tuning properties via strong FS. The second subnetwork is intracortical levels of processing within output layers, which further modulates information from input layers by strong SS and sends computational results from this cortical area to other areas. The two subnetworks across layers may provide general computation for all primary sensory cortices because the circuitry within V1 is comparable with other primary sensory cortices (Lund, 1988; Linden and Schreiner, 2003).

### Differences between the current study and earlier work

In an earlier study, we demonstrated dynamic responses to stimulus orientations (similar to Fig. 1*B*) and proposed a three-component model with two types of suppression (Xing et al., 2005). However, the present study is the first to report a complete picture of dynamic laminar processing of orientation information and laminar distribution of suppressive components. Furthermore, the current study also demonstrated a laminar-specific relationship between V1 suppression and two important functional properties, orientation selectivity and surround suppression. The results described above represent new findings that distinguish the current study from our previous studies (Xing et al., 2005, 2011). Because of the limited number of single neurons (i.e., SUA), earlier studies were not able to demonstrate such laminar patterns. By using simultaneously recorded MUAs, the current study substantially increased the number of sites for each V1 depth and the statistical power for detecting laminar patterns. Another unique feature of the current study is that we drove all V1 layers using gratings with the same spatial frequency, which was optimized for input layer 4Cα (2 cycles/degree). In contrast, in our earlier work, we drove each recorded site using its own optimal spatial frequency, which varied between 0.1 and 10 cycles/deg (Xing et al., 2005). Differences in stimulus parameters are crucial for studying laminar processing. Our earlier work demonstrated that some single neurons exhibited orientation tuning with a “Mexican hat” shape (Xing et al., 2005). In contrast, in the current dataset from awake monkeys, we seldom observed this tuning pattern, possibly because of the differences between optimizing spatial frequency for each single neuron versus fixing spatial frequency for all recorded sites. Cells or sites with “Mexican hat” tuning typically exhibit an optimal spatial frequency higher than the spatial frequency we used in this study. It may be valuable for future studies to investigate the laminar processing of spatial frequency in V1 layers. Another potentially important difference is that our earlier studies were based on data from anesthetized monkeys, whereas the current results were based on awake monkey data.
